# Supplementary material for: Content-rich biological network constructed by mining PubMed abstracts
Source: BMC Bioinformatics. 2004 Oct 8;5:147. doi: 10.1186/1471-2105-5-147 (PMC528731; doi:10.1186/1471-2105-5-147)
Supplement: Additional File 2 — The original results of the above study (non-essential files are deleted to keep the file size under the limit set by BMC bioinformatics). [file 1471-2105-5-147-S2.bz2 › chilibotAdditionalFile2/dip05/147ID9139681E523/html/ATP1A1_ATP1B1.html]

 


 **ATP1A1** and **ATP1B1** 
  
Found 1 abstracts in PubMed, retrieved 1.  
 

 What does Google say? 
 PDF only 
| .edu only 

---

**Interactive relationship** (e.g. stimulation, inhibition, etc)

**Non-interactive relationship** (e.g. studied together, co-existance, homology, etc.)

- In the case of prime clone analyses using the long PCR experiments, the distance between ATP1a  [ **ATP1A1** ]  and ATP1b  [ **ATP1B1** ]  or ATP1b  [ **ATP1B1** ]  and ATP1c was approximately 10 kb or 7 kb, respectively.  Ref: 10407267 Yeast, 1999
- and c long PCR analyses of prime clones 70113 and 70804, both of which contained two ATP1 gene copies, ATP1a  [ **ATP1A1** ]  and ATP1b  [ **ATP1B1** ] , and ATP1b  [ **ATP1B1** ]  and ATP1c, respectively, using 30 nucleotides just inside the 3 end sense and 5 end antisense of the ATP1 coding region as primers.  Ref: 10407267 Yeast, 1999
